# Systematic evaluation of multiple NGS platforms for structural variants detection

**DOI:** 10.1016/j.jbc.2023.105436

**Published:** 2023-11-07

**Authors:** Xuan Meng, Miao Wang, Mingjie Luo, Lei Sun, Qin Yan, Yongfeng Liu

**Affiliations:** 1 School of Medicine, Southern University of Science and Technology, Shenzhen, China; 2 Research Cooperation Department, GeneMind Biosciences Company Limited, Shenzhen, China

**Keywords:** structural variations (SV), NA12878, WGS, next-generation sequencing, GenoLab M

## Abstract

Structural variations (SV) are critical genome changes affecting human diseases. Although many hybridization-based methods exist, evaluating SVs through next-generation sequencing (NGS) data is still necessary for broader research exploration. Here, we comprehensively compared the performance of 16 SV callers and multiple NGS platforms using NA12878 whole genome sequencing (WGS) datasets. The results indicated that several SV callers performed well relatively, such as Manta, GRIDSS, LUMPY, TARDIS, FermiKit, and Wham. Meanwhile, all NGS platforms have a similar performance using a single software. Additionally, we found that the source of undetected SVs was mostly from long reads datasets, therefore, the more appropriate strategy for accurate SV detection will be an integration of long and shorter reads in the future. At present, in the period of NGS as a mainstream method in bioinformatics, our study would provide helpful and comprehensive guidelines for specific categories of SV research.

Structural variations (SVs) are typically defined as genomic alterations larger than 50 bp in length (1), such as aberrations that change the size, location, orientation, copy number, and sequence content (2). SV occurs in many subtypes, including deletions (DELs), duplications (DUPs), insertions (INSs), inversions (INVs), translocations (TRAs), and other rearrangements (3, 4). SVs are present in approximately 1.5% of the whole human genome (5). Some SVs that cause great changes in gene structure or expression are the drivers of many inherited human genetic diseases, such as cancer (6), autism (7), and Parkison’s disease (8). Therefore, SV research has become an important topic in genetic studies (3).

The detection of SVs has originated with the technology progression. Commonly used classic SV detection methods are FISH for single gene SV changes and array-based technologies (SNP array, array CGH, or CMA) for whole genome level SV changes (9, 10, 11, 12). However, they can not detect inversions or balanced translocations (13). During the past 2 decades, Next-generation sequencing (NGS) gained world scientific attention due to its high throughput and wide application in healthcare (14). Whole-genome sequencing (WGS) and whole-exome sequencing (WES) are common sequencing strategies in inherited genetic disorders, while WGS has a high coverage of the human genome. Hence, WGS has emerged as a comprehensive way of diagnosing genetic diseases. Besides primary variant calling and short InDel calculation, specific algorithms are designed for SV detection using WGS data (15). These algorithms can be classified into four categories based on their calculation logic: read pair calling, read depth calling, split read calling, and *de novo* assembly calling (16, 17, 18, 19). Each algorithm has its specific advantages for SV calling. We chose 16 commonly used SV callers because they were highly cited and represented a cross-section of calculation logic. Besides, they can detect SVs based on a single WGS data but do not require matched datasets.

Illumina's bridge amplification-based sequencing has led the NGS market for over a decade, owing to its high efficiency and quality (20). MGI DNBSEQ platforms have attracted more attention recently due to their comparable sequencing results at low instrumental and reagent costs (21). Two years ago, GeneMind Biosciences launched the GenoLab M platform based on its own sequencing-by-synthesis technique. The new sequencing platform has shown its equivalent ability compared to Illumina platforms in detecting gene expression and LncRNA in RNA sequencing (22), whole genome bisulfite sequencing (23), spatial transcriptomics (24), metagenomic next-generation sequencing (25), as well as detecting SNP and InDels in WGS (26).

This study comprehensively compared SV detection of the standard cell line NA12878 WGS data produced by the four NGS platforms *via* 16 popular SV callers. We benchmarked available SV callers based on WGS to determine the efficacy of available tools and explored a good balance between sensitivity and precision on multiple NGS platforms.

## Results

### SV detection based on WGS of multiple platforms

**Figure 1:**
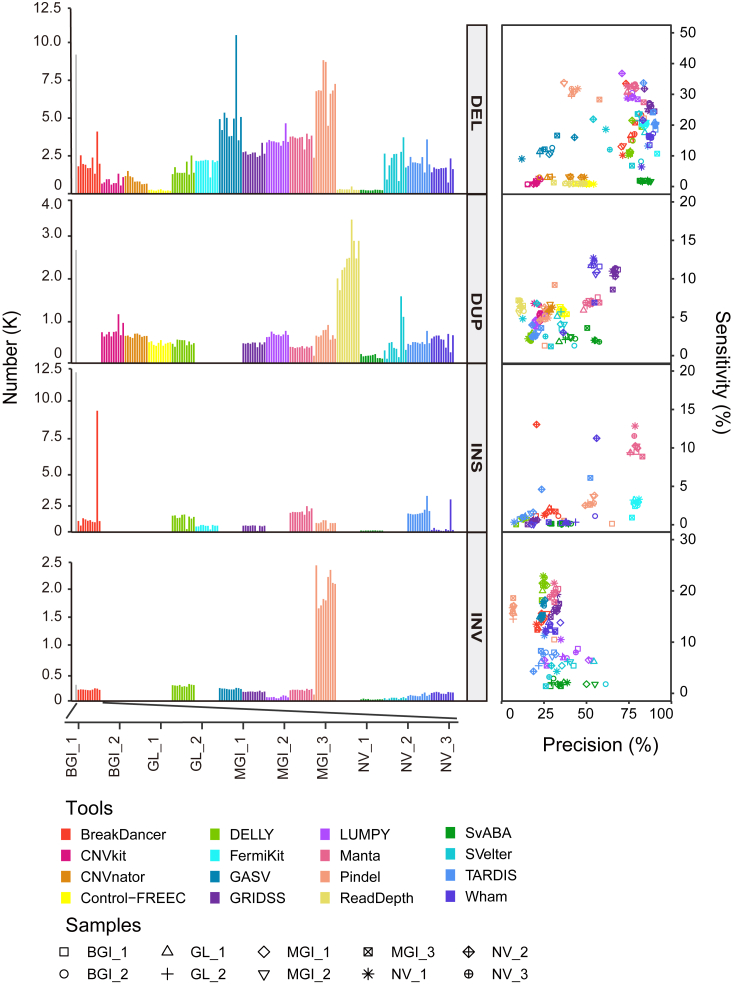
**Comparison of inferred SVs across 16 SV callers and platforms on four SV categories of NA12878 data.** (*Left*) Bar plots depict the numbers of SV detected across SV callers. The *blank fields* indicated that the tools don’t support the SV category. The *gray bar* represents the number of valid benchmarks for each SV category. (*Right*) Evaluation of the four SV categories from all SV datasets on the benchmark. Dot plots show the precision and recall of all SV datasets across SV callers. 16 SV callers were marked with different colors, and different datasets were marked with different point types. Abbreviations of four SV categories: BGI, BGISEQ-500; DEL, Deletion; DUP, Duplication; GL, GenoLab M; INS, Insertion; INV, Inversion; MGI, MGISEQ-2000; NV, NovaSeq 6000.

We detected SVs on WGS datasets of NA12878 under an average depth of 30 (Table S1). Among the four categories, the number of DELs variants (average 2202) was the most, while only a quarter of the true sets. Pindel and GASV detected the most DELs (mean±SD, 6382 ± 1248 and 5043 ± 953, respectively), and Control-FREEC (181 ± 30) had the fewest number (Fig. 1). GRIDSS, TARDIS, and Wham showed higher precision (average 93.24%, 91.04%, and 90.50%, respectively), with low sensitivity (26.43%, 21.55%, and 15.53%, respectively). Manta, LUMPY, and GRIDSS had the highest F1-score (45.47%, 43.28%, and 40.97%, respectively) in Table S2. Meanwhile, there were consistent results on sequencing platforms in these three tools: 45.44 ± 1.41% by Manta, 43.14 ± 1.19% by LUMPY, and 40.87 ± 1.73% by GRIDSS (Fig. 1). For duplication variants, three tools (BreakDancer, FermiKit, and GASV) could not support. The number of deletion variants by ReadDepth (2341 ± 433) was closest to the true sets (2607). Meanwhile, GRIDSS and Wham achieved fine performances with higher precision (68.44% and 53.21%), while the sensitivity (∼10%) and F1-scores (∼20%) were relatively low. Regarding insertion variants, seven tools failed to detect them, and the gap between the detected INSs and the true set was the largest. Manta detected the most insertion variants with high platform consistency. Besides, Manta has the highest precision (81.94%) and sensitivity (10.24%) across all datasets for insertion type. The number of inversion variants (average 284 INVs) was the fewest, while the number of inversion variants was the closest to the true sets (274). GRIDSS and Manta performed the highest precision (30.40% and 29.00%) and sensitivity (16.88% and 19.60%) than other tools with F1-score >20%. The results indicated that the distribution is relatively wide in detecting different categories of SV by various tools. Some software detect SVs with apparent false positives, such as Pindel in INVs calling. So, we explored the consistent trend across sequencing platforms and software. It revealed that the sequencing platforms have less effect than tools (Fig. S1). Overall, software Manta and GRIDSS performed better in detecting multiple SV types for WGS datasets.

### Comparing consensus of SV detection in NGS platforms

**Figure 2:**
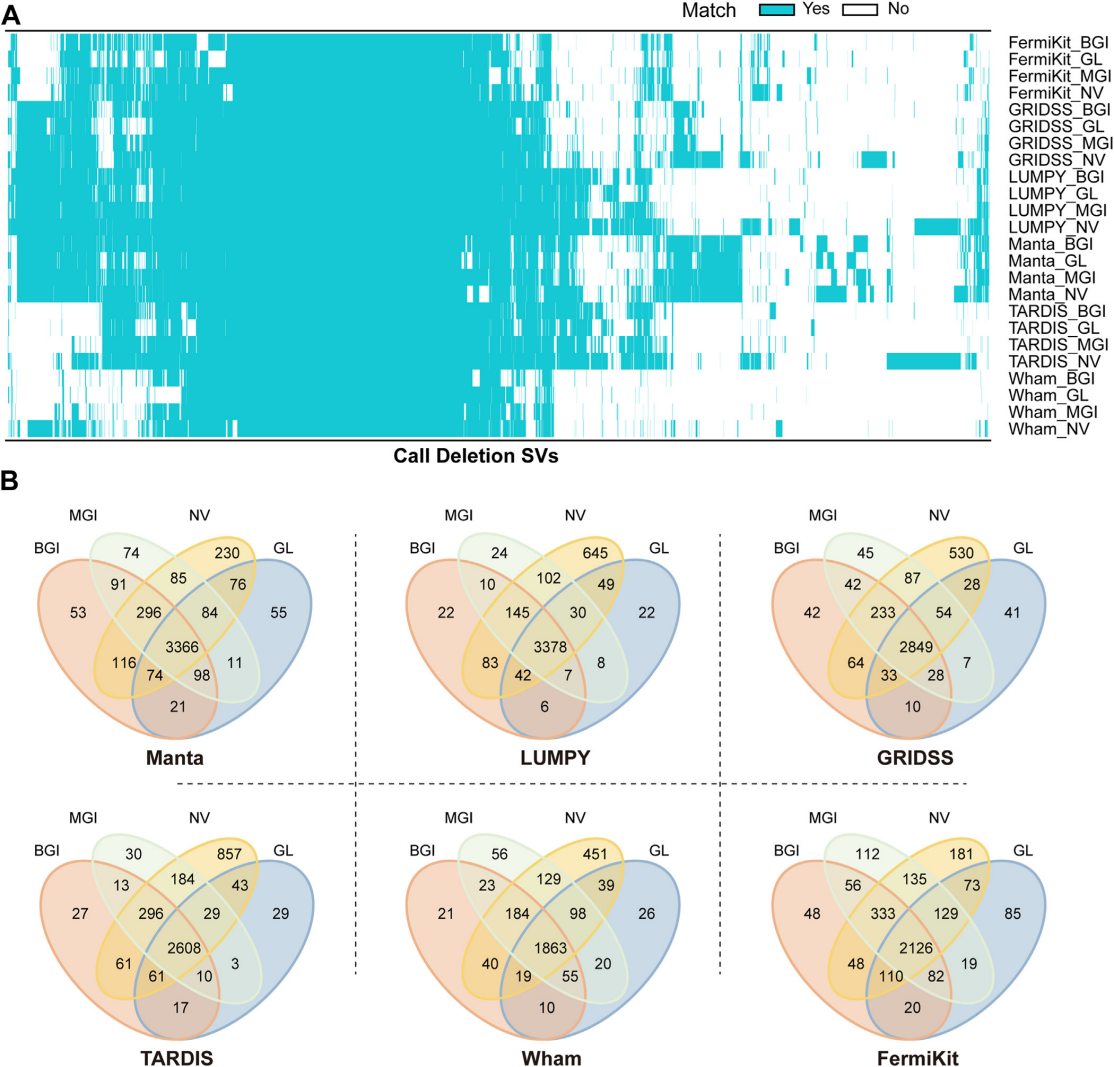
**Consensus true deletions heatmap and Venn of different NGS platforms using different SV callers.***A*, heatmap showed the called deletion variants overlap with the true sets on the four platforms by six SV callers, including Manta, LUMPY, GRIDSS, TARDIS, Wham, and FermiKit. The horizontal axis represented the all-called true positive deletion sets. *Each row* represented the results of one platform by one SV caller. The *blue color* denoted that the given deletion variant was called by the SV caller on the platform. The blank field indicated the opposite. *B*, Venn diagrams displayed the consistency of true positive deletion variants detected on four NGS platforms by each SV caller. Abbreviations of four platforms: BGI, BGISEQ-500; GL, GenoLab M; MGI, MGISEQ-2000; NV, NovaSeq 6000.

We evaluated SV consensus among 16 callers in four platforms based on the benchmark of NA12878, which mainly combined the Database of Genomic Variants data (DGV, based on NGS), with the PacBio SV data generated from the assembly of long reads (16). The number of DELs, DUPs, INSs, and INVs were 2392 (74.1% of truth sets), 1108 (57.5%), 7324 (46.4%), and 128 (55.9%), respectively (Figs. 1 and S2). The results revealed that the DELs detected were more comprehensive than other types, and the percentage of DELs supported by multiple SV callers simultaneously was the highest. In the following evaluation, we selected six tools (FermiKit, GRIDSS, LUMPY, Manta, TARDIS, and Wham) with high F1-score relatively. Then, we assessed the performance of four platforms with respect to DELs calling *via* six SV callers. The results indicate that the NovaSeq 6000 platform detects the most DELs, the BGISEQ-500 and MGISEQ-2000 platforms have similar detection numbers, and the GenoLab M platform detects the least (Fig. 2*A*). In terms of tools, the top three were Manta, LUMPY, and GRIDSS, with Wham detecting the fewest DELs. Arts of Words, NovaSeq 6000 platform combined with Manta can call the most DELs. So, the consistency of true positive DELs among sequencing platforms was further analyzed one by one tool. Manta and LUMPY had the largest common DELs (71.2% and 73.9%), and unique DELs set on NovaSeq 6000 was the most (Fig. 2*B*).

Next, we compared the ratio of false-negative (FN) deletions in the coverage aspect on multiple platforms. The coverage regions (≥90%) of FN deletions were more than 83.47% (average 87.18 ± 3.71), and further more than 74.36% (average 79.41 ± 5.05) that meet the depth requirement (reads supported ≥4) in Fig. S3. In source aspects by platforms and SV callers, it found a significantly lower proportion of NGS source than of PacBio SV source by these six different SV callers (*p* value ranged from 5.4e-08–3.6e-16 with *t* test), and a significant differences of the ratio by four platforms (*p* value ranged from 4.6e-13–1.4e-09 with *t* test) as shown in Fig. S3. The FN deletions ratio of NGS sources was highly similar (*p* = 0.97 with ANOVA), and Pacbio SV sources have a similar median (0.94 ± 0.004) across all platforms. These results revealed that the coverage of the whole genome was very high *via* NGS, and the FN SVs were mainly owing to long reads strategies.

### Performance of length distribution for deletions

**Figure 3:**
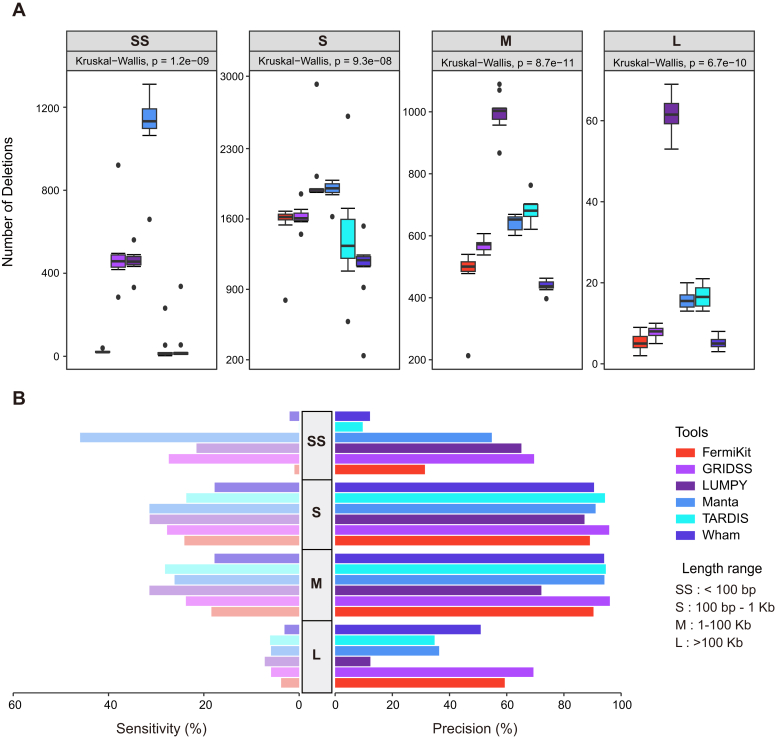
**Comparison of size range distribution for deletions detected across six SV callers and platforms**. *A*, the number of deletions detected on different length distributions of each caller. *B*, the precision and sensitivity of each size range of deletions were determined. Size range: SS, <100 bp; S, 100 bp to 1 Kb; M, 1 to 100 Kb; L, >100 Kb. Six callers were marked with different colors.

Since each SV caller was designed for different types, we assessed the performance of the size range distribution (<100 bp as SS, 100 bp-1 Kb as S, 1–1000 Kb as M, and >1000 Kb as L) for DELs. It revealed that a few callers exhibited limits in a specific size range (Figs. 3 and S4). For example, FermiKit, TARDIS, and Wham barely detected the SS type. In all size ranges, there were significant differences among all tools (Kruskal-Wallis test, *p* < 9.3e-08). Manta had the highest number for SS type, and LUMPY and Manta could detect the most DELs for S type. LUMPY had absolute superiority for M and L types. Meanwhile, the precision and sensitivity of the deletions were calculated in each size range based on the benchmark. For the SS type, precision and sensitivity were uneven across all callers. Especially, Manta had a notable advantage, although its sensitivity was only 45.96%. For M and S types, except for FermiKit and LUMPY, the other tools performed high precision (>90%) with slightly lower sensitivity, possibly owing to the undetected deletions in the benchmark being from PacBio SV data. For L type, awful performances indicated that these six tools were not suitable for large size deletions. Furthermore, we evaluated the true positive (TP) deletions and found that NovaSeq 6000 platform detected the most TP deletions in all types, followed by MGISEQ-2000 and GenoLab M (Fig. S4). Overall, LUMPY, Manta, TARDIS, and GRIDSS performed similarly in the M, and S types, and Manta performed best in SS type deletions.

### Run time and memory performance

**Figure 4:**
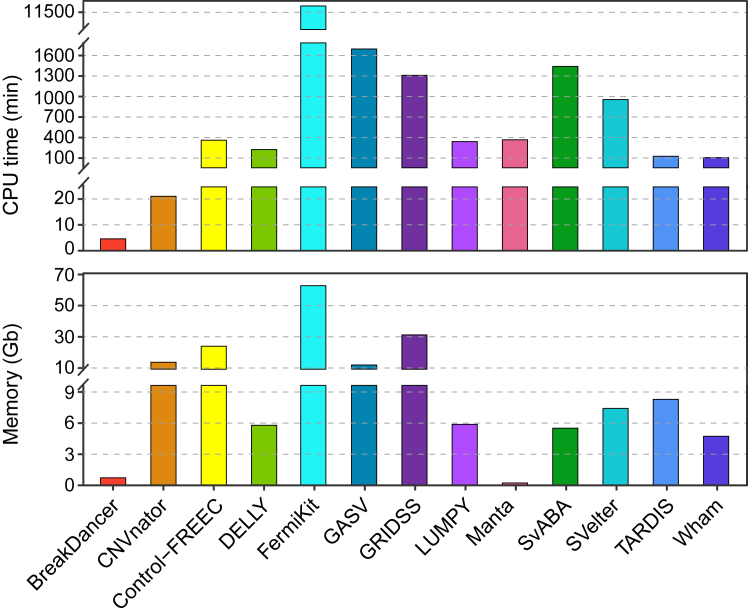
**Run time and maximum memory consumption for SV callers**.

Furthermore, we compared the CPU time and the maximum memory across all SV callers. A single CPU was used on each caller. The run time varied widely with more than three orders of magnitude (Fig. 4). BreakDancer took the least time and also the smallest memory. Among these six tools, exhibiting good calling accuracy in this study, TARDIS and Wham required a shorter time, and Manta consumed the fewest memory. FermiKit took the longest run time and the largest memory to perform the analysis.

## Discussion

SVs are important variants for the whole human genome, detecting which in sequencing data is crucial for genetic diseases and healthcare-related analysis (27). With the increasing development of NGS technology, the sequencing efficiency is getting higher and the sequencing cost is getting lower. SV detection based on WGS has been extensively researched and applied. Multiple platforms confirmed their ability to generate sequencing data suitable for calculating SVs. Meanwhile, more NGS platforms were released and provided more options for genome research. In this study, our data were from multiple NGS platforms (NovaSeq 6000, BGISEQ-500, GenoLab M, and MGISEQ-2000) of the NA12878 cell line. The sensitivity and accuracy of SV detection greatly influenced the following analysis. We selected 16 SV callers with a higher citation rate to detect germline SV based on NGS data, for SV detection to perform a comprehensive benchmarking.

There is a great variance in detecting tools for SVs (16). Knowing the advantages and limitations of each caller is critical to selecting proper tools for detecting interest SVs (16, 28, 29). We used an SV reference set (9223 DELs, 2607 DUPs, 13,669 INSs, and 290 INVs) as an available benchmark (16). We compared the sensitivity and precision of SV variants across multiple platforms and tools under default parameters. Manta, LUMPY, and GRIDSS exhibited the highest F1-score for DELs calling. For DUPs, GRIDSS, Wham, and Manta showed high precision. Manta exhibited the best performance for INSs events, while, GRIDSS and Manta showed the highest precision and sensitivity for INVs calling on all platforms. These tools perform differently in SV detection, which differences in design background and calculation could cause. Meanwhile, the performance of SV quantity and F1-score were remarkably similar among all sequencing platforms. These results indicated that the sequencing platforms have less effect than callers. We identified several SV tools that had performed well on benchmarks, such as Manta and GRIDSS performed better in detecting multiple SV events for WGS datasets.

Although several reference sets of NA12878 have been published, there are still no recognized SV sets as a gold standard dataset (30, 31, 32). The reference SV dataset was mainly derived from the DGV and PacBio SV data (16). It was currently the most comprehensive SV dataset. In this study, we determined the distribution of SV detection based on multiple NGS platforms by various software, nevertheless, there were quite a bit SVs that were not called, unfortunately. In addition, some false-positive variants leading to a decrease in accuracy, especially for DUPs and INVs. Except for the limitations of callers, it would in part be due to imperfections in the NA12878 reference dataset. In spite of these shortcomings, SV evaluation on multiple software or platforms was much more meaningful in selecting a suitable SV caller or sequencing platform in the current research (16). The deletion detected was more comprehensive than the other events for comparison with a reference set, so only the DELs were explored in depth. The performance of SV distribution exhibited high consistency across all platforms, consistent with the true positive analysis of deletions.

Although the regions of FN variants had high coverage by NGS reads, and had been undetected by all the callers unfortunately. First, SVs were genomic variants larger than 50 bp (even several megabytes) in length and may include multiple genes. However, the reads produced by NGS sequencers were usually shorter and below 300 bp (33). Long reads are more beneficial for SV detection. So, the NGS reads put forward a high requirement of recognition algorithm for breakpoints. Second, each software’s algorithm has some limitations to meet its design purpose (29). Third, SVs were more complex structural variations than SNP or InDel. GRCh37 is a recognized reference for the human genome, however, there are still some gaps (34). Moreover, the human reference has some repetitive sequences (35). These increase the difficulty of identifying the breakpoints of structural variations, especially DELs, INVs, and INSs, including more complex inter-chromosomal translocation and complex variants (16). Building a more accurate SV reference set, involving experimental validation is very important. Furthermore, improving the algorithm of the available software and developing new software based on NGS platforms would improve the detection rate of SVs. We also found that the proportion of FN variants of DGV was significantly lower than PacBio SV for all tools. Obviously, the main source of FN variants was long-read data. Long-read sequencing technologies, namely Pacific Biosciences sequencers and Oxford Nanopore Technologies sequencers, have enabled the precise detection of long SVs (36), including long insertions by transposable elements, such as LINE-1 (37). Because long reads overcome the limitations of NGS (38), the sensitivity of SVs of NGS platforms would be improved by adding long-read sequencing platforms.

The size range of SV is an essential factor in measuring the accuracy of SV detection. We compared the performance of several major well-performing software (Manta, GRIDSS, LUMPY, TARDIS, FermiKit, and Wham) in DELs detection. Some SVs were undetected in the benchmark set by all tools. This study illustrated that the main possible reasons were the long reads source of the true set and the limitation of the SV caller's algorithm. In size ranges of between 100 bp to 100 Kb, the six tools achieved higher performance. However, there was uneven performance in the shorter size and larger size regions, especially in size <100 bp. Meanwhile, NovaSeq 6000 and MGISEQ-2000 were better than other platforms on the TP deletions detected in all the SV ranges. In addition, there were significant differences in required maximum memory and run times resources. BreakDancer took the least run time and Manta consumed the fewest memory, while FermiKit required 2555 and 68 times more than BreakDancer and Manta, respectively.

To summarize, we evaluated the SV detection ability in WGS datasets across multiple platforms and found that the precision and recall of SVs detection were not higher than SNP, consistent with previous findings (26). A fundamental limitation was the need for more well-defined SV datasets, especially the somatic sets and more complex structure rearrangement types. On the other hand, a tool usually presents the best performance in a particular size range or a special type (16). Thus, to obtain the expected SV results, a suitable algorithm should be selected that fits the type and size range. It remained a great challenge to improve SV detection capabilities for developers. With the rapid development of sequencing technology, the future of the SV algorithm was likely to combine NGS and long reads (39). In addition, of all the datasets, we noted that the average length of insert size of MGI_3 was less than 300 bp (262 bp), and the others were greater than 369 bp (from 369 to 575 bp). However, no significant differences in SV results were found. It indicated that the SV detection could be compared within a certain size range of insert fragments. Furthermore, several SV callers performed well on NA12878 in this study, such as Manta, GRIDSS, LUMPY, TARDIS, FermiKit, and Wham. One of our following research plans is whether they are suitable to perform well on other samples (such as tumor samples) or other NGS platforms (such as Ultima Genomics or Element Bio). Overall, our study provides a comprehensive guide for SV detection on the NGS platform.

## Experimental procedures

### Data acquirement and primary process

Ten WGS datasets were adopted in this study. Nine WGS FASTQ raw datasets of NA12878 on BGISEQ-500, MGISEQ-2000, GenoLab M, and NovaSeq 6000 were downloaded from the China National GeneBank Sequence Archive (CNSA) and National Center for Biotechnology Information (NCBI). Besides, we constructed one library and sequencing on GenoLab M, referring to the method in previous research (30). All raw datasets were pair-end (PE) reads in the FASTQ format. The insert size of the WGS library was about 400 bp. The data were dissected into an average depth of 30× for WGS *via* in-house script. The sequencing adapters and low-quality reads were filtered and trimmed by FASTP v0.20.0 (40) with default parameters.

### SV callers and detected pipeline

There were more than 70 published SV callers based on WGS datasets now. 16 popular SV callers were selected, which were widely used and had a high citation rate. They were BreakDancer v1.3.6 (41), DELLY v1.0.3 (42), GRIDSS v2.13.1 to 0 (43), Manta v1.5.1 (44), Pindel v0.2.5 (45), SVelter v1.1.2 (46), TARDIS v1.0.8 (47), Wham v1.7.0 (48), SvABA v1.1.3 (49), LUMPY v 0.2.13 (50), CNVnator v0.4.1 (51), Control-FREEC v11.6 (52), CNVkit v0.9.10 (53), ReadDepth v0.9.8.4 (54), FermiKit v0.13 (55) and GASV v1.4 (56). Nine tools can not detect all SV subtypes (Table S3).

The filtered reads were aligned to the human genome (GRCh37) by “Sentieon BWA” of Sentieon software v202112.04 (57) and sorted by the “sort” utility tool. Then “LocusCollector” and “Dedup” tools were employed to remove duplicate reads, and the re-duplicated BAM files were obtained. Quality metrics reports were generated by Qualimap BamQC v2.2.1 (58) for the BAM files. Next, we used SV callers with default parameters to detect SVs based on each bam file, and expected FermiKit based on FastQ files. We converted all SV sets to VCF format to deal with the following processing conveniently. All the SV sets were annotated using Annovar (59) to perceive the functional consequences of the gene.

### Evaluation of the SVs calling

For the reference sets of NA12878, we used the available benchmarks for evaluating SVs, including 9233 deletions, 2607 duplications, 290 inversions, and 13,669 insertions in one study reported in Journal Genome Biology (16). To reduce the false positive, the SV results were filtered according to the following criteria (1): supporting read pairs<4 (2), overlapping a gap in the reference genome, and (3) not autosomal or chrX. The statistical method for true positive (TP) and false negative (FN) variants was referred to in the previous study (16). To evaluate accuracy of SVs, the following formulas were used.

• Sensitivity = TP/(TP + FN).
• Precision = TP/(TP + FP).
• F1-score = 2∗Sensitivity∗Precision/(Sensitivity + Precision).

### Comparison of SVs among multiple platforms and multiple tools

We calculated precision and sensitivity based on each SV set. We further merged the SV variants of all datasets using a single software to access the performances of all SV callers. The different performance among multiple platforms was evaluated using deletion variants detected across six tools with higher F1-score. Finally, we divided the SVs into four categories according to the length of SVs detected for comparing differences of all platforms and tools. All statistical tests were performed in R (version 4.1.2). The *t* test and ANOVA test were used to compare.

## Data availability

The reads files of WGS are available in CNGB Sequence Archive (https://db.cngb.org/cnsa/) under project accession number CNP0003843.

## Supporting information

This article contains supporting information (22, 27, 60, 61).

## Conflict of interest

The authors declare that they have no conflicts of interest with the contents of this article.
