# Supplementary material for: Systematic evaluation of multiple NGS platforms for structural variants detection
Source: J Biol Chem. 2023 Nov 7;299(12):105436. doi: 10.1016/j.jbc.2023.105436 (PMC10724692; doi:10.1016/j.jbc.2023.105436)
Supplement: Supporting information [file mmc1.docx]

**Supplementary Data**

**Title**

Systematic evaluation of multiple NGS platforms for structural variants detection

**Running title**: Structural variants detection in multiple platforms

**Authors:**

Xuan Meng

MiaoWang

Mingjie Luo

Lei Sun

Qin Yan

Yongfeng Liu

Correspondence author: Yongfeng Liu liuyongfeng@genemind.com.

Address: 5F and 6F, Block 2, Luohu Investment Holding Building, No. 116, Qingshuihe 1st Road, Luohu District, Shenzhen, Guangdong, 518000, China

**Contents**

Table S1. Whole-genome sequencing data of NA12878 based on different platforms.

Table S2 SV calling results for all WGS datasets.

Table S3. The list of SV callers used in this study.

Figure S1. The Heatmap of consistency ratios in pairwise comparisons across all SV datasets.

Figure S2. Heatmap showed the called SVs overlap with the true sets across all SV callers in 10 datasets.

Figure S3. Comparison of false negative deletions in the coverage and source on multiple NGS platforms.

Figure S4. Performance of the size range distribution for true deletions called on multiple sequencing platforms.

**The PDF file includes:**

Figures S1 to S4

**Other Supplementary Material for this manuscript includes the following:**

Table S3

Supporting tables

**Table S1. Whole-genome sequencing data of NA12878 based on different platforms.**

| **Dataset Name** | **Platform** | **Accession Number** | **Database** | **Reference** | **Data Size (Gb)** | **Depth (X)** |
| --- | --- | --- | --- | --- | --- | --- |
| BGI_1 | BGISEQ-500 | CNP0000042-CNR0001437 | CNGB Sequence Archive | (1) | 93.92 | 29.72 |
| BGI_2 | BGISEQ-500 | CNP0000042-CNR0001438 | CNGB Sequence Archive | (1) | 93.94 | 29.72 |
| GL_1 | GenoLab M | CNP0002694-CNR0501869 | CNGB Sequence Archive | (2) | 97.84 | 30.84 |
| GL_2 | GenoLab M | CNP0003843-CNR0657526 | CNGB Sequence Archive | this study | 96.62 | 30.46 |
| MGI_1 | MGISEQ-2000 | CNP0000059-CNR0028192 | CNGB Sequence Archive | (1) | 95.23 | 29.45 |
| MGI_2 | MGISEQ-2000 | CNP0000059-CNR0028193 | CNGB Sequence Archive | (1) | 95.53 | 29.50 |
| MGI_3 | MGISEQ-2000 | CNP0000813 | CNGB Sequence Archive | (3) | 99.31 | 31.32 |
| NV_1 | NovaSeq 6000 | CNP0000042-CNR0001439 | CNGB Sequence Archive | (1) | 92.07 | 28.85 |
| NV_2 | NovaSeq 6000 | SRR8454589 | NCBI Sequence Archive | (4) | 98.03 | 30.58 |
| NV_3 | NovaSeq 6000 | CNP0002694-CNR0501871 | CNGB Sequence Archive | (2) | 97.33 | 30.59 |

**Note:**

1. CNGB, China National GeneBank DataBase

**Table S3. The list of SV callers used in this study.**

| ID | **Tools** | **SV_Types** | | | | **Literature Citations** | **Version** |
| --- | --- | --- | --- | --- | --- | --- | --- |
| 1 | GRIDSS | DEL | DUP | INS | INV | 259 | v2.13.1 |
| 2 | Manta | DEL | DUP | INS | INV | 1,338 | v1.5.1 |
| 3 | Pindel | DEL | DUP | INS | INV | 2,121 | v0.2.5 |
| 4 | SVelter | DEL | DUP | INS | INV | 47 | v1.1.2 |
| 5 | TARDIS | DEL | DUP | INS | INV | 57 | v1.0.8 |
| 6 | Wham | DEL | DUP | INS | INV | 110 | v1.7.0 |
| 7 | DELLY | DEL | DUP | INS | INV | 1,808 | v1.0.3 |
| 8 | SvABA | DEL | DUP | NA | INV | 288 | v1.1.3 |
| 9 | LUMPY | DEL | DUP | NA | INV | 1,273 | v 0.2.13 |
| 10 | BreakDancer | DEL | NA | INS | INV | 1,573 | v1.3.6 |
| 11 | CNVnator | DEL | DUP | NA | NA | 1,538 | v0.4.1 |
| 12 | Control-FREEC | DEL | DUP | NA | NA | 860 | v11.6 |
| 13 | CNVkit | DEL | DUP | NA | NA | 1,270 | v0.9.10 |
| 14 | ReadDepth | DEL | DUP | NA | NA | 260 | v0.9.8.4 |
| 15 | FermiKit | DEL | NA | INS | NA | 109 | v0.13 |
| 16 | GASV | DEL | NA | NA | INV | 169 | v1.4 |

**Note:** The statisticaldate of Literature Citations was September 25, 2023

**Supporting figures**


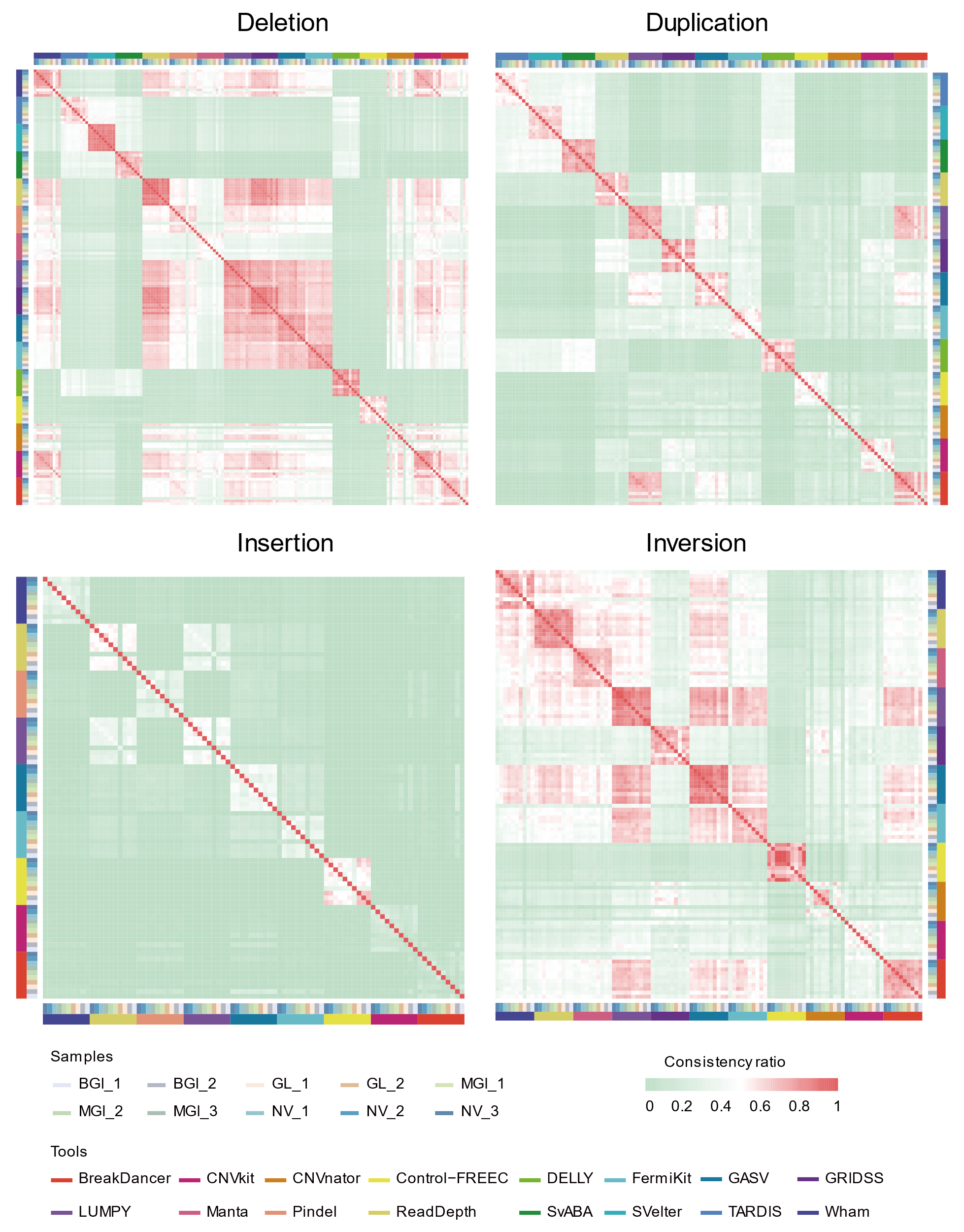


**Figure S1. The Heatmap of consistency ratios in pairwise comparisons across all SV datasets.**


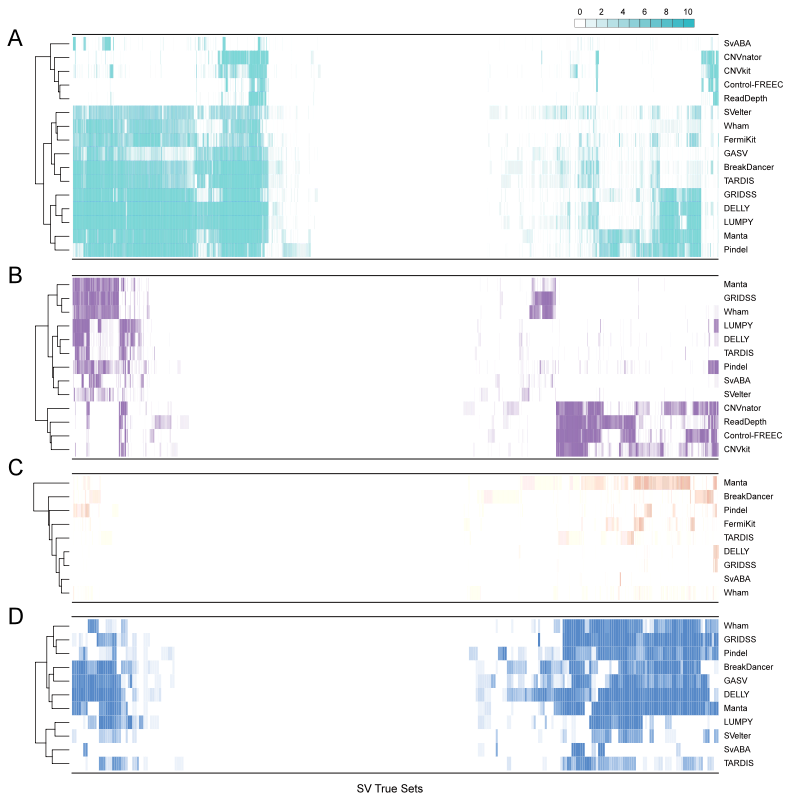


**Figure S2. Heatmap showed the called SVs overlap with the true sets across all SV callers in 10 datasets.** (A) Deletions across 16 tools (true sets: n=9233) (B) Duplications across 13 tools (true sets: n=2607) (C) Insertions across nine tools (true sets: n=13,669) (D) Inversions across 11 tools (true sets: n=290). The horizontal axis represented all true sets of SV, and the different colors indicated different SV types. Each row represented the SV caller. The gradient colors indicated the number of detected SV. The blank field indicated that the [corresponding](javascript:;) tool did not detect the SV in the ten datasets.


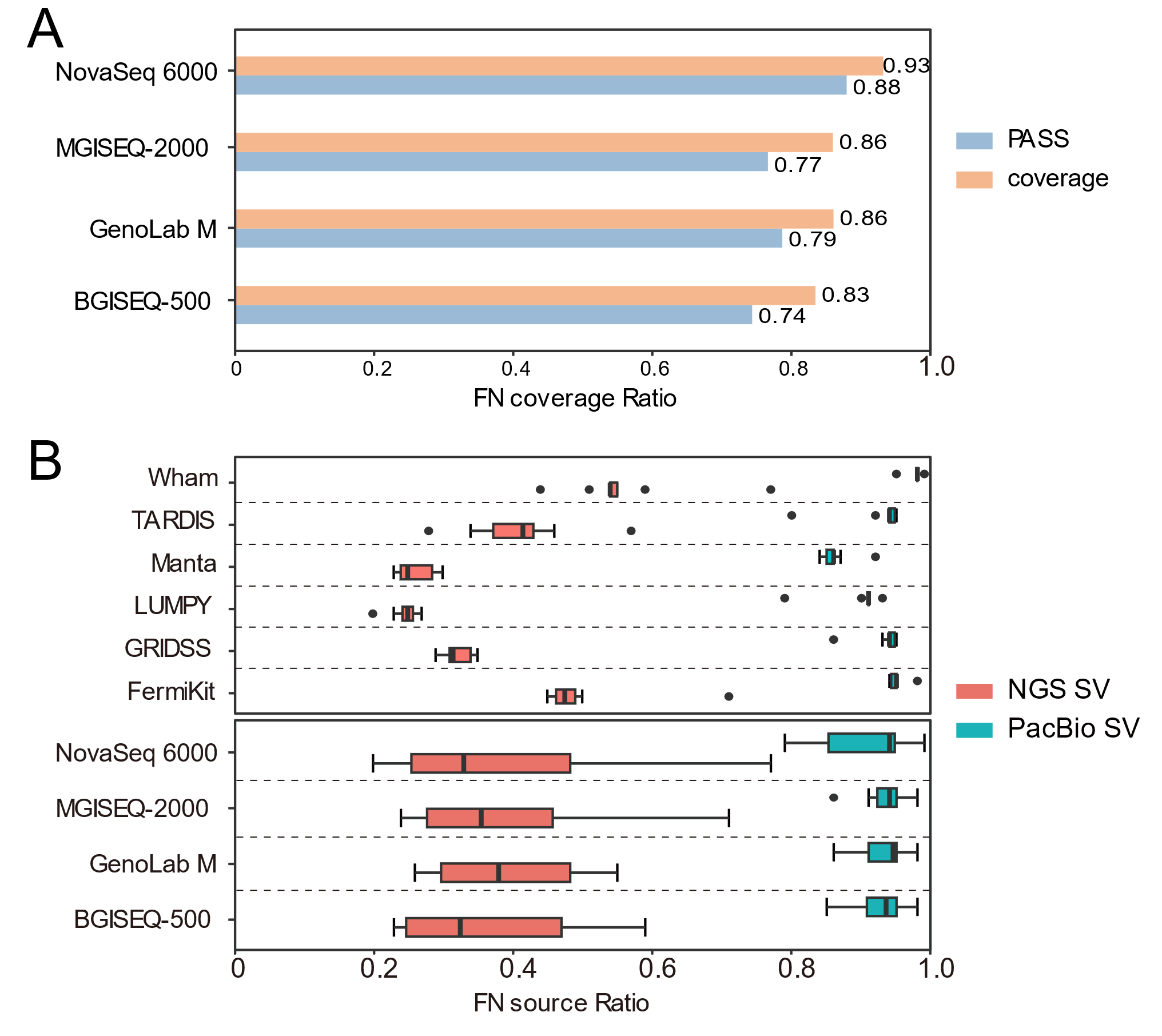


**Figure S3. Comparison of false negative deletions in the coverage and source on multiple NGS platforms.** (A) The coverage ratio on multiple NGS platforms.Orange box represented the average proportion of FN deletions with coverage (≥ 90%) based on reads alignment results. Blue box plot represented the proportion of FN deletions that pass the standards: coverage(≥ 90%) and depth (≥ 4*n, n was the number of samples on the corresponding platform). (B) The source ratio on multiple NGS platforms or multiple tools. Green box plot and red box plot represented the proportion of FN deletions come from PacBio SV data and NGS data, respectively.


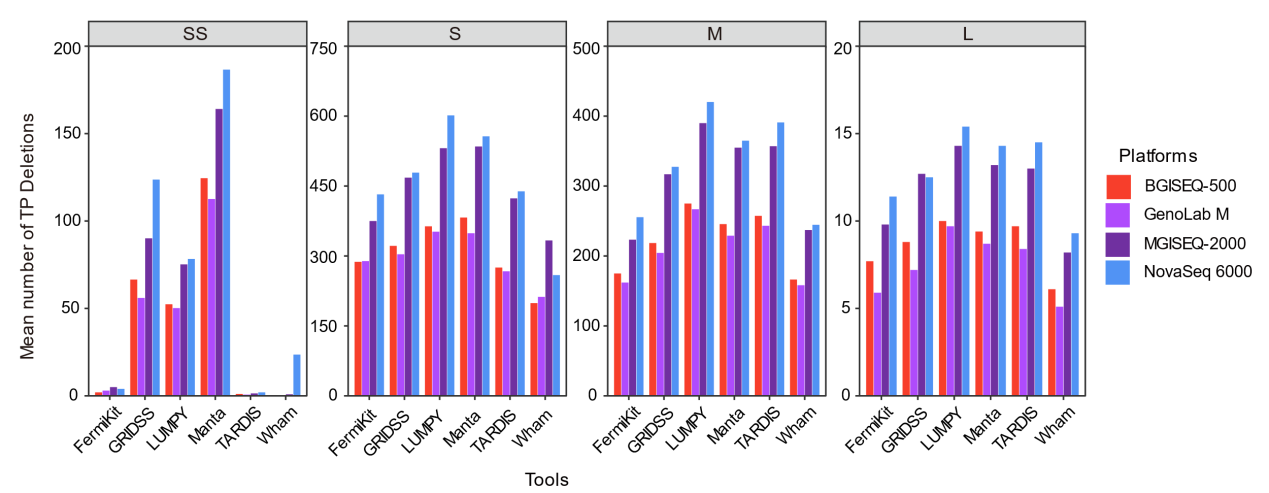


**Figure S4. Performance of the size range distribution for true deletions called on multiple sequencing platforms.** The colors represent different platforms. The horizontal groups were each SV caller.

**Supporting Reference**

1. Rao, J., Peng, L., Liang, X., Jiang, H., Geng, C., Zhao, X., Liu, X., Fan, G., Chen, F., and Mu, F. (2020) Performance of copy number variants detection based on whole-genome sequencing by DNBSEQ platforms. *BMC bioinformatics* **21**, 1-14

2. Liu, Y., Han, R., Zhou, L., Luo, M., Zeng, L., Zhao, X., Ma, Y., Zhou, Z., and Sun, L. (2021) Comparative performance of the GenoLab M and NovaSeq 6000 sequencing platforms for transcriptome and LncRNA analysis. *BMC genomics* **22**, 1-12

3. Xu, Y., Lin, Z., Tang, C., Tang, Y., Cai, Y., Zhong, H., Wang, X., Zhang, W., Xu, C., and Wang, J. (2019) A new massively parallel nanoball sequencing platform for whole exome research. *BMC bioinformatics* **20**, 1-9

4. Chen, J., Li, X., Zhong, H., Meng, Y., and Du, H. (2019) Systematic comparison of germline variant calling pipelines cross multiple next-generation sequencers. *Scientific reports* **9**, 9345
